# Greener, Energy-Efficient and Sustainable Networks: State-Of-The-Art and New Trends

**DOI:** 10.3390/s19224864

**Published:** 2019-11-08

**Authors:** Josip Lorincz, Antonio Capone, Jinsong Wu

**Affiliations:** 1 Department of electronics and computing, Faculty of electrical engineering, mechanical engineering and naval architecture (FESB), University of Split, 21000 Split, Croatia; 2 Department of electronics, informatics and bioengineering, Politecnico di Milano, 20133 Milan, Italy; antonio.capone@polimi.it; 3 Department of electrical engineering, Universidad de Chile, Santiago 8370451, Chile; wujs@ieee.org

**Keywords:** energy-efficiency, wireless, green, sustainable, data centre, networks, ICT, 5G, power, wired access, IoT

## Abstract

Although information and communications technologies (ICTs) have the potential of enabling powerful social, economic and environmental benefits, ICT systems give a non-negligible contribution to world electricity consumption and carbon dioxide (CO_2_) footprint. This contribution will sustain since the increased demand for user′s connectivity and an explosion of traffic volumes necessitate continuous expansion of current ICTs services and deployment of new infrastructures and technologies which must ensure the expected user experiences and performance. In this paper, analyses of costs for the global annual energy consumption of telecommunication networks, estimation of ICT sector CO_2_ footprint contribution and predictions of energy consumption of all connected user-related devices and equipment in the period 2011–2030 are presented. Since presented estimations of network energy consumption trends for main communication sectors by 2030 shows that highest contribution to global energy consumption will come from wireless access networks and data centres (DCs), the rest of the paper analyses technologies and concepts which can contribute to the energy-efficiency improvements of these two sectors. More specifically, different paradigms for wireless access networks such as millimetre-wave communications, Long-Term Evolution in unlicensed spectrum, ultra-dense heterogeneous networks, device-to-device communications and massive multiple-input multiple-output communications have been analysed as possible technologies for improvement of wireless networks energy efficiency. Additionally, approaches related to the DC resource management, DCs power management, green DC monitoring and thermal management in DCs have been discussed as promising approaches to improvement of DC power usage efficiency. For each of analysed technologies, future research challenges and open issues have been summarised and discussed. Lastly, an overview of the accepted papers in the Special Issue dedicated to the green, energy-efficient and sustainable networks is presented.

## 1. Introduction

**Figure 1:**
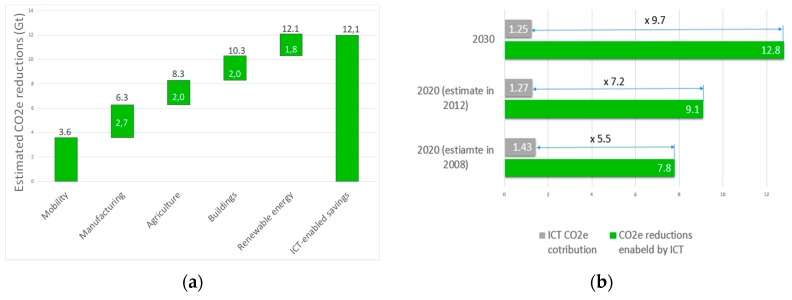
Estimated: (**a**) contribution of different industry sectors to global carbon-dioxide equivalent (CO_2e_) reduction by 2030 [1], (**b**) information and communications technology (ICT) sector CO_2e_ “footprint” contribution and enabled reductions to global CO_2e_ emissions expressed in Gt [2].

United Nations (UN) General Assembly have set sustainable development goals (SDGs) by the year 2030, and analyses presented in [1] show that information and communications technologies (ICTs) have the potential of enabling powerful social, economic and environmental benefits. However, a lack of exploration and innovation attempts dedicated to the search for answers on how SDGs can be achieved through the implementation of ICT, requests for more global governmental, technological, scientific and industrial attempts for accomplishing UN SDGs. The role of ICTs is twofold; while ICTs and networking currently contribute non-negligibly to the global energy consumption and carbon dioxide (CO_2_) emissions, they will also contribute to the reduction of carbon dioxide (CO_2_) and energy consumption of other industry sectors. This unique position of the ICT sector is confirmed in the SMARTer2030 report of the Global e-Sustainability Initiative (GeSI) [2], according to which expected carbon-dioxide equivalent (CO_2e_) emissions of the ICT sector in 2030 can be kept at the same level as those in 2015. This means that ICTs will yield the 20% reduction of global CO_2e_ emissions by 2030 (Figure 1a). To illustrate the importance of ICTs in reducing CO_2e_ emissions, it is worth to state that contribution to CO_2e_ reduction due to the deployment of renewable energy sources by 2030 is estimated on 10.3 Gt, which is a (for 1.8 Gt) lower contribution to CO_2e_ reductions when compared with 12.1 Gt of estimated CO_2e_ reduction yield by the ICT sector (Figure 1a).

According to estimates of GeSI, the ICT sector will give by 2030 significantly higher contribution to CO_2e_ emission reductions when compared with other industry sectors (e.g., mobility, manufacturing, agriculture, buildings, etc.). To achieve such CO_2e_ emission reductions until 2030, a significant decrease of ICT sector CO_2e_ emissions in global CO_2e_ emissions are envisioned by 2030 [2]. Based on results presented in SMARTer2030 report (Figure 1b), in 2020 ICT sector’s CO_2_ emissions “footprint” is estimated on 2.7% (1.43 Gt) of global CO_2e_ emissions, while due to expected improvements in energy efficiency of ICT systems, ICT sector will in 2030 contribute with 1.97% (1.25 Gt) to global CO_2_ emissions. This means that estimated CO_2e_ emissions avoided by the use of ICT systems in 2030 (12.08 Gt) will be 9.7 times higher than the CO_2e_ emissions generated by implementing the same ICT systems (Figure 1b). Thus, an expected increase in the implementation of ICT systems in the future can potentially alleviate the need for selection among environmental protection and economic prosperity and it can pave the way to the achievement of both goals.

Despite such positive estimates, the increased demand for user′s connectivity and an explosion of traffic volumes necessitate continuous expansion of current ICTs services and deployment of new infrastructures and technologies which must ensure the expected user experiences and performance.

**Figure 2:**
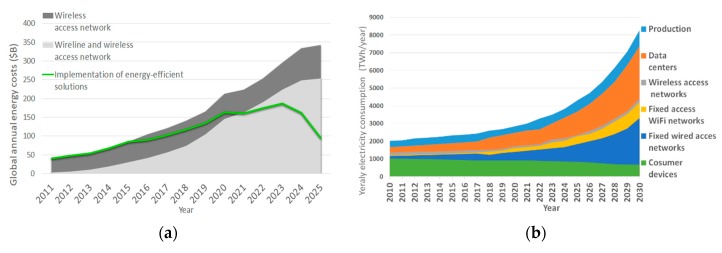
Estimation of (**a**) costs for the global annual energy consumption of telecommunication networks in period 2011–2025 [2], (**b**) expected total annual energy consumption per different ICT systems in period 2010–2030 [10].

This results in an increase in the energy consumption and energy cost of the ICTs infrastructure, which in recent years become one of the major concerns for the ICT sector. Due to the expected increase in diversity of connected objects, devices, applications and services and because of the rapid growth of the worldwide broadband subscribers, predictions related to global annual monetary costs for the energy consumption of ICT infrastructure are worrying. The energy consumption estimated for wireline (access, metro, edge, core networks and the associated data centres) and wireless access networks is presented in Figure 2a [3]. According to these forecasts, if no energy-efficiency improvements will be implemented, monetary costs for the global annual energy consumption of telecommunication networks will raise 8.6 times, more specifically form $40 billion in 2011 to $343 billion in 2025. This increase of energy consumption costs is a direct consequence of the need for satisfying explosive growth of annual global internet protocol (IP) traffic, which is estimated on 4.8 ZB/year by 2022, or 396 EB/month. In 2022 this will result in a threefold monthly increase of IP traffic since 2017 (122 EB/month), or an astonishing 14.1 times increase since 2011 (28 EB/month) [4,5].

Due to increased energy costs pushed by constantly increasing traffic volumes, current network energy costs of telecommunication service operators in developing countries already span between 40% and 50% of provider operational expenditures (OPEX), and between 7% and 15% of the OPEX for operators in developed countries [6,7,8]. This is confirmed by some telecom operators which start reporting energy bills of up to $1 billion, while some expect to reach these costs by 2020 [9].

High energy costs of telecommunication networks presented in Figure 2a correlate with estimations of energy consumption trends of different ICT systems presented in Figure 2b. Estimates presented for the period 2011–2030 are performed with an assumption that takes into account expected annual: future improvements in the energy efficiency of ICTs systems, trends in future IP traffic growth and future improvements in electricity usage per traffic unit [10]. According to estimations presented in Figure 2b, expected annual electricity consumption of consumer devices (including desktop, monitor, laptops, televisions (TVs) and peripherals, tablets, mobile phones, smartphones, modems, etc.) will contribute to the global electricity consumption of ICT systems by 2030 with 8.1% (670 TWh). Estimations further assume for fixed wired (core, distribution and access) networks, WiFi networks (consumer premises WiFi equipment), radio part of the wireless access network (second (2G)/third (3G)/fourth (4G)/fifth generation (5G)) and data centres (servers, power supply and cooling elements), yearly energy consumption contribution to the annual electricity footprint of ICT systems equal to 31.95% (2641 TWh), 10.75% (889 TWh), 2.35% (195 TWh) and 35.89% (2967 TWh), respectively (Figure 2b). Additionally, estimates for annual electrical energy consumed for the production of different ICT devices (user, wired and wireless network equipment, data centre devices) are anticipated at 10.92% (903 TWh) of total ICT energy consumption by 2030 (Figure 2b).

Moreover, best, expected and worst-case forecasts related to the overall yearly electricity footprint of ICT systems in 2030 equals to 2698, 8265 and 30,715 TWh, respectively, which means that energy consumption impact of ICT systems for the overall global annual energy consumption can be, in the best-case, equal to 8%, or 21% for the expected (Figure 2b) and even an astonishing 51% for the worst estimation case. To get a sense of the rapidness of ICT energy consumption increase, in 2012 it was estimated that the complete ICT sector contributes approximately 6% to global electricity consumption [11]. Hence, worst or even expected (Figure 2a) forecasts of ICT energy footprint trends in global annual energy consumption by 2030 are alarming. This dramatic increase in energy consumption of ICT systems justifies the precipice of economic unsustainability. Obviously, current technology improvements cannot cope with the increasing energy consumption of the ICT sector and it is imperative to find novel solutions that will alleviate this problem.

The rest of the paper is organised as follows. The energy consumption of user-related devices is analysed in Section 2. Section 3 and Section 4 give an overview of research challenges for energy-efficiency improvements of radio access networks and data centres, respectively. A short description of all articles accepted for publication in the Special issue on green energy-efficient and sustainable networks of the Sensors journal are presented in Section 5. Finally, some concluding remarks are given in Section 6.

## 2. Energy Consumption of User-Related Devices

**Figure 3:**
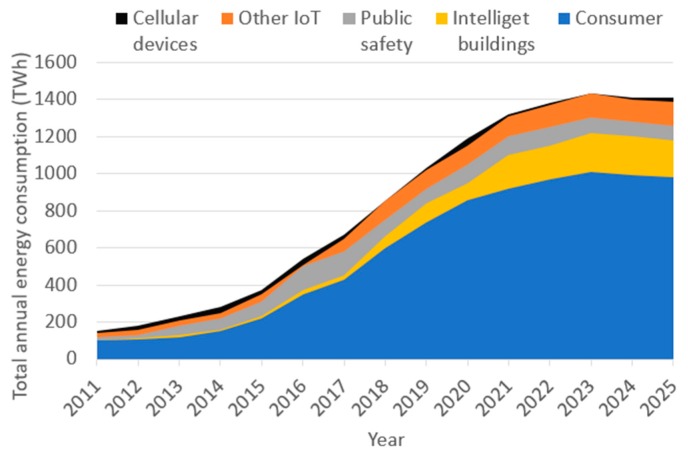
Estimations of energy consumption of all connected user-related devices and equipment for the period 2011–2025 [3].

According to presented in the previous section, the energy consumption of data centres (DCs) and communication network devices is just one part of the overall ICT energy consumption, while energy consumption of user-related devices presents the other part. The energy consumption patterns of user-related devices point to different challenges and require different approaches to energy consumption reductions, than those envisioned for network and DC devices. Energy consumption estimates of user-related devices for the period 2011–2025 are presented in Figure 3 [3]. Presented estimates are performed for all connected user devices in cellular networks, internet of things (IoT) applications, public safety, intelligent buildings and generally for all consumer devices with a network connection. Estimates consider the explosive growth of user-related devices from about 50 billion in 2011 to 110 billion devices connected to the network in 2025 [3]. Forecasts for the global energy consumption of these user-related devices estimate the energy consumption raise from about 180 TWh in 2011 to 1400 TWh in 2025 (Figure 3), which represents a 7.7 time increase in the period of one and a half-decade.

Obviously, this estimated energy consumption increase is unacceptable, and attempts focused on alleviating such trends must take into account specific peculiarities of user-related devices. For example, a single sensor or IoT device, in reality, consume rather low amounts of energy in absolute values, however, it is expected that a vast number of such devices will be installed worldwide. On the other hand, battery-less user-related devices must have constant power supply while battery-powered devices must have a periodic power supply for battery recharging. This power supply can be obtained from the electricity grid, by means of renewable energy sources, by means of energy harvested from the environment or through the combination of these power sources. Hence, the problem related to the energy footprint of user devices is not solely related to their annual energy consumption trends, it is also related to the sources of energy supply and energy autonomy in the case of battery-powered devices. Some estimates show that the number of user devices powered by rechargeable, grid, network and renewable sources will increase in the period from 2011 to 2025 for 13×, 54×, 380× and 378× times, respectively [3]. Such figures mandate a necessity for significantly higher usage of renewable energy sources.

Additionally, advances in battery storage, new solutions for lowering power consumption of user devices and relying on energy harvesting is another possibility for energy footprint improvements. This will be especially important since running power lines to a huge number of user devices or repeatedly change of batteries will not be viable from the practical or economic point of view. Hence, implementation of a fully connected world characterized by IoT and internet of everything (IoE) applications will not be possible on a large scale unless the energy supply challenges of user devices are properly solved. Future solutions must offer trade-off in the energy equation among better energy storage, more effective use of harvested and renewable energy sources and lowering power consumption of user devices.

### Energy Consumption Trends

**Figure 4:**
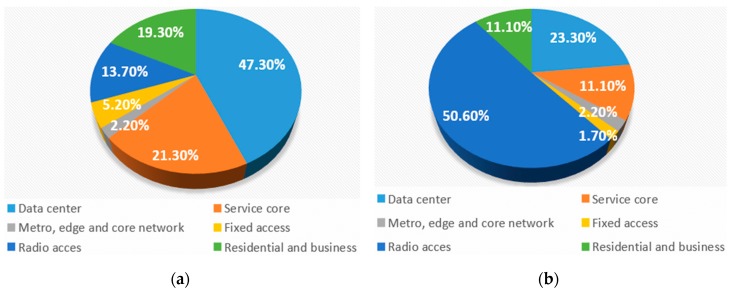
Estimated network energy consumption for main communication sectors in: (**a**) 2013 and (**b**) 2025 [3].

Although Figure 2a,b shows estimated trends for telecommunication networks in terms of expected total annual monetary costs and electricity consumption per different ICT systems, more detail analyses are presented in this section in order to understand the future trends in energy consumption of communication networks. In Figure 4, energy consumption is breakdown into six main network sectors, more specifically: edge and core networks, radio access, DCs, service core, fixed access and residential and businesses. Contribution to the total annual energy consumption of each network sector in 2013 and estimates for 2025 are presented in Figure 4a,b [3], respectively. Estimations are performed based on expected IP traffic growth and by assuming the potential benefits of new network architectures and technologies. According to Figure 4b, energy consumption will remain high or even increase in two sectors: the data (cloud) centres and the wireless radio access network, while in other sectors energy consumption will remain or even decrease. However, different technology improvements are required in each of these sectors to ensure that an increase in IP traffic in the future can be supported in an economically viable and sustainable way by 2025. Since wireless radio access networks and data centre sectors are the highest contributors to the overall network energy consumption, the next sections are dedicated to the presentation of main research challenges related to the improvement of energy efficiency (EE) of these sectors.

## 3. Research Challenges for Energy-Efficiency Improvements of Radio Access Networks

**Figure 5:**
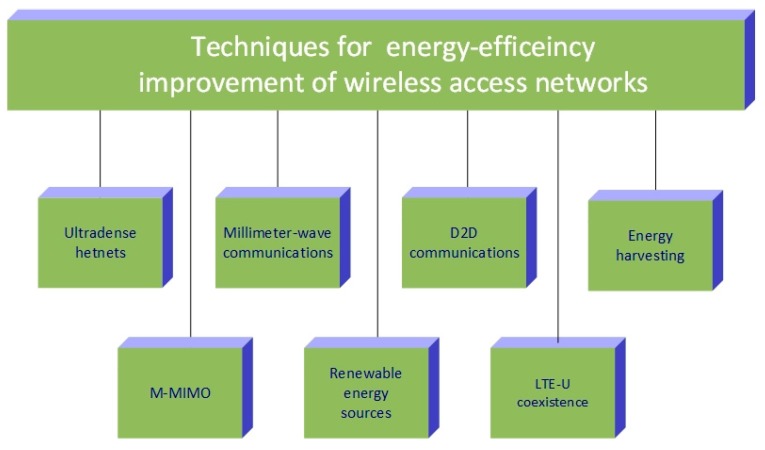
Techniques for energy-efficiency improvement of radio access networks.

In this section, a review of the last research activities on green radio access approaches and energy harvesting for the power supply of network devices in cellular access networks is presented. Also, potential technical demands and some research topics for realizing green, energy-efficient and sustainable radio access networks are emphasized. For 5G networks, as currently the most prominent wireless network technology, tremendous performance improvements are envisioned. These improvements encompass support of: a thousand-fold increase in throughput in comparison to present networks, up to ca. 7.6 billion mobile subscribers with the connection of at least 100 billion devices worldwide, up to 10 Gb/s individual user broadband speeds, IoE communications, tactile Internet applications and the network latency of 1 ms or lower. To satisfy such demanding performance gains, different novel technologies are emerging, but performance improvements incurred by 5G networks do not come without drawbacks. One of the major consequences is the degradation of EE expressed in bits/Joule (b/J), which has been broadly accepted as the EE metric for wireless communication systems [12]. It is expressed as

(1) $$EE=\frac{FR\;\times \;SS\;\times \;BW\;\times \;log_{2}\left(1+SINR\left(D\right)\right)}{P_{c}+P_{T}}\;\left[b/J\right],$$

where *SS*, *BW* (Hz), *FR*, *D* (m), *P_C_* (W) and *P_T_* (W) represents the number of spatial streams (spatial multiplexing factor), the bandwidth of signal, frequency reuse factor, distance among communicating devices, circuit (mostly static) and transmit (mostly dynamic) power consumption of communicating devices, respectively. According to EE Equation (1), the EE of cellular networks can be increased by augmenting the signal bandwidth, the multiplexing factor, the frequency reuse factor, or by lowering the circuit and transmit power consumption. In this regard, different paradigms for 5G networks have emerged (Figure 5): Communications based on millimetre-waves (mmWave), long term evolution in unlicensed spectrum (LTE-U), ultra-dense heterogeneous networks (UDNs HetNets), device-to-device (D2D) communications and massive multiple-input multiple-output (M-MIMO) communications. The impact of each technology on EE of radio access networks is further discussed. In Table 1, an overview of technologies for EE improvements of wireless networks with future research challenges characteristic for each technology is summarised.

### 3.1. Ultra-Dense Heterogeneous Networks

In essence, UDNs are heterogeneous networks based on a massive deployment of diverse types of base stations (BSs), where macro-cells (of macro BSs) ensure base signalling coverage while micro-cells (of mini/micro/pico/femto BSs) fulfils the demand for high throughput [13,14]. Such broadly accepted radio access network architecture based on decoupling data and signalling contributes to EE improvement of cellular networks and enables separation of downlink and uplink [15] communications. Due to the reduction of distance between users terminals (UTs) and BSs accomplished with densification of BSs allocation in such heterogeneous networks (HetNets), the EE improvements of the network are reflected in a significant reduction of transmit powers and consequently energy consumption for both (UT and BS) transceivers. Also, signalling and data decupling enable replacement of the macro-cell BSs by more energy-efficient types of BSs having distant radio access unit′s (RAUs) controlled from central location without impacting the small-cell BSs layer. Additionally, decoupling enables combining different radio access technologies (RATs) such as mmWave and WiFi in existing networks, which can help in achieving further EE gains. Moreover, the separation of uplink and downlink transmission enables versatile association schemes among UTs ad BSs, which also can lead to significant energy savings for both, BSs and UTs [12]. Nevertheless, UDN concept is not without drawbacks. It is expected that the realization of such HetNets requests additional equipment and BS sites that will increase telecom operators’ (TOs) total network energy consumption for up to 150%–170% by 2026 [16]. Hence, novel approaches to energy control within both, the 5G network infrastructure and changes in the way TOs purchase and deliver electricity to 5G networks will become critical as they extend density, coverage and capacity over the next decades. Also, signalling and data decupling raise the complexity of HetNets management and contribute to a significant increase in signalling overheads (Table 1). This request further investigations in the development of new signalling and network designs, which will enable full exploiting of signalling and data decoupling while preserving network EE.

Another important approach to improvement of BS energy-efficiency is concept based on on/off switching of BSs (i.e., BS sleeping) to save energy [17,18,19]. Applicability of this concept is related to the nature of wireless traffic loads which varies in time and space. This concept enables shutting down or putting into sleep mode some BSs in periods of low traffic loads and activation of BSs when there is a need for satisfying increased traffic demands. Such dynamic management of BSs activity in the radio access networks enables tuning of BS power consumption according to real traffic variations, which eliminates the waste of energy imposed with the traditional concept based on BSs which are permanently active, even in the periods with low or without any user traffic [12]. However, ensuring full-service area coverage, signalling for smooth user handovers among BSs and elimination of overloading of those BSs that remains active is a challenging task in case of BS on/off deployments (Table 1). To ensure optimal balance between network EE and service quality, further improvements of radio resource management algorithms must be developed and implemented.

Due to the high-frequency reuse factor, inter-cell interference represents another challenge to EE implementation of ultra-dense HetNets. The Tx power increase of two neighbouring BSs initiated in process of BS radio resource management, can have a negative impact in terms of signal cancelling caused by inter-cell interference [20]. This degrades the system throughput and consequently leads to lowering of network energy-efficiency. Although complete elimination of inter-cell interference is not possible, management schemes which suppress interference, such as cooperative transmission, smart power control, interference alignment and resource scheduling and partitioning are needed for the successful proliferation of energy-efficient ultra-dense HetNets (Table 1).

### 3.2. Massive-MIMO Technology

Since it is based on exploiting a large number of BS antennas for serving many users with the same time-frequency resources, M-MIMO concept significantly improves multiplexing and array gain of 5G transmission systems. The drawback of M-MIMO implementation is that such concept increases significantly power consumption of individual BS sites. When compared with 4G BSs, M-MIMO strongly contributes to increase of 5G BSs power consumption due to the increase in a number of analogue-to-digital converters with corresponding digital circuitry and power amplifiers needed for M-MIMO operation [21]. More specifically, typical 4G BS contain four transmit (Tx) and receive (Rx) elements (in so-called 4 × 4 MIMO arrays configuration), while 5G BSs are intended to work in up to 64 × 64 configurations, which is the reason why it is expected that 5G BS will have three times higher power consumption than its 4G predecessor. On the other hand, M-MIMO can bring some advantages with respect to EE of wireless networks [12]. This is because the uplink Tx power of single-antenna UT can be proportionally reduced with the number of MIMO BS antennas in the case when the equivalent results as those of a related single-input single-output transceiver wants to be achieved [22]. However, only reducing the Tx power of UTs is not sufficient for significant improvements of EE in wireless networks, since power consumed by electronic circuits has linear growth with the number of MIMO signal processing circuits, which has a non-negligible impact on the overall power consumption (Table 1). Hence, determining an optimal number of antennas in M-MIMO systems arises as important research topic which generally yields assumption according to which, a larger number of antennas must be deployed in systems which Tx power dominates in the overall power consumption, and vice versa [14].

Furthermore, M-MIMO systems with a large number of antennas installed enable the implementation of simpler precoding algorithms and signal detection and transmission at the BS, which further enable significant savings in power consumption contributed by BS hardware. In comparison with the implementation of existing signal processing methods (successive cancelling of interference and dirty paper coding), implementation of advanced algorithms for signal processing such as maximum ratio transmission/combining contributes to the reduction of the dissipated energy required for signal processing computations [12]. Additionally, since M-MIMO systems demand much smaller RF Tx power (of the order of milliwatts), power amplifier losses during operations will be reduced which can bring significant power savings. Nevertheless, major challenges requesting broad investigations are currently present in the design of UTs hardware (Table 1). Major performance bottlenecks related to UT hardware are the limited physical size of UTs, lacking space for implementation of a large number of antennas and demanding requirements on battery depletion.

Above all, M-MIMO systems require accurate and timely channel state information′s (CSIs) which acquisition is directly related to the Tx antenna number. This leads to the significant power consumption of pilot subcarriers and new approaches such as semi-orthogonal pilot design and pilot beamforming needs further exploiting in order to reduce the contribution of pilot transmission to the overall M-MIMO system energy consumption. Additionally, pilot interference incurred by reusing the same resources of pilots in neighbouring cells of multi-cell locations also diminishes the EE of M-MIMO systems (Table 1). Hence, designing pilot interference mitigation approaches as well as balance in the exploitation of resources in time and frequency for training of pilots in downlink and uplink, are important topics that must be slaved in order to reach high EE of M-MIMO systems.

### 3.3. Millimetre-Wave Communications

For transmission in the mmWave spectrum, the conventional transceiver architecture having each antenna connected to the corresponding radio-frequency (RF) chain is energy-inefficient [12]. Inefficiency is a consequence of huge power consumption which emerges from the concurrent processing of vast amounts of data burst Giga-samples/s per each RF chain. Thus, an approach to alleviate the power consumption problem is to implement both, the digital and analogue beamforming, where every RF chain can be connected to all (fully controlled architecture) or to some antennas (partially controlled architecture) of a transmission system. The signal phase of each antenna must then be scheduled by a network of digital and analogue phase shifters (PSs) [23]. The fully connected architecture demands hundreds or even thousands of PSs, which maximizes spatial degrees transmissions utilization and minimizes EE. The partially controlled architecture exploits only a limited number of PSs that improve system EE, but reduces spatial degrees freedom and consequently transmission rates. Possible solutions, which are currently a field of research, aim to find optimal hybrid control architectures (Table 1). These architectures are based on a different number of antennas and RF chains, combining and precoding approaches, Tx power allocations and antenna arrays having a lens with energy-focusing. The development of these architectures can jointly or separately optimize the system performance whit minimal impact on EE degradation [24].

Besides the high-power demands of a huge number of PSs, another power consumption problem characteristic for mmWave systems are analogue-to-digital converters (ADCs). The power dissipation of ADCs increases exponentially with the increase in the number of bits per sample and linearly with the augmentation of the sampling rate [12]. Additionally, the data circuits which connect the digital elements to the ADCs are high energy consumers and have an evident correlation with the adopted sampling rate resolution (Table 1). This motivates the search for finding optimal ADCs in terms of sampling rate resolution, which will efficiently balance between the power consumption and the data rate or ensure optimal combining of low and high-resolution ADCs in order to maximize EE.

### 3.4. Renewable Energy Sources

An approach based on powering BSs sites using energy harvested from renewable energy sources such as wind, solar, fuel cell or combination of these energy sources significantly contributes to the improvement of wireless network EE. Current trends in terms of integration of renewable energy into power supply systems of contemporary wireless networks are twofold. The first approach is dedicated to the replacement of an off-grid diesel-based BS power supply system with those relying solely on some renewable energy sources. The other approach is based on the so-called hybrid BSs sites which use different renewable energy sources or a mix of renewable, diesel generator and/or grid energy. In addition to EE improvements and operational expenditure reductions, such approaches significantly reduce or even completely eliminate diesel generator CO_2_ emissions from BS sites [25,26]. However, the optimal selection of renewable energy sources in terms of size and power generation capacity, for the specific site remains one of the major challenges (Table 1). Hence, further investigations in the development of simulation tools that can fairly estimate the techno-economic aspect of transforming a typical BS site in green BS site must take place.

Additionally, the integration of renewable energy sources into BSs power supply systems can provide compensation for the additional circuit power consumption in case of installing more BSs on BS site [12]. Also, dense allocation of BSs employing energy harvesting from renewable sources can facilitate possible energy cooperation between BSs. This cooperation can be based on transferring through power lines superfluous energy collected on sites harvesting more energy, to BSs sites that harvest less energy (Table 1).

However, the major challenge in realization of durable BS site power supply solutions can be found in the intermittent nature of renewable energy sources, limited battery capacities installed on sites and necessity for ensuring stable and without any interruptions power supply of BSs sites. This imposes the development of resource allocation algorithms for the management of BS site power demand. Such algorithms must consider the traffic variations and wireless channel state information′s, power supply impacted with the unpredictable nature of renewable energy sources and battery recharging and depletion cycles [27]. Algorithms for efficient energy flow management of BS sites are generally categorized as offline and online algorithms. The first one can be developed by exploiting optimization theory approaches and the second one assumes that some statistical data is accessible at the Tx side or they use the insights observed from the offline algorithm. Since 5G networks are characterised with very dynamic traffic variations, results of offline algorithms often serve as performance upper bounds for online algorithms. Nevertheless, the development of an optimal resource allocation algorithm for a specific hybrid BS power supply solution, continues to be an object of research interest (Table 1).

### 3.5. Device-To-Device Communications

This type of communication offers effective local spectrum reuse through two modes of operation: the cellular mode where UTs communicate via BSs and the D2D mode which ensure possible communication of UTs directly with each other [28]. D2D mode of communication can be realised through reuse of the spectrum portions that have not been assigned (known as overlay communication) or has already been scheduled to UTs (known as underlay communication). Overlay D2D communication does not generate co-channel interference, which results in more efficient spectral efficiency (SE) of the D2D system [12]. In periods when such interference is weak, it is possible to switch to underlay communication which offers more energy-efficient D2D communication system design (Table 1). However, for switching among underlay and overlay communication designs, effective algorithms must be envisioned, what represents a prominent research field.

Another advantage of D2D communications is the ability of proactive cooperation between users, what can bring EE improvements in 5G networks, particularly in terms of extending the mobile devices’ battery lifetime. More specifically, active UTs in D2D networks can work as mobile relays or cluster heads of UT clusters and local cashing devices, and each of these working modes can bring possible EE improvements of the cellular network [12]. A mobile relay mode based on a multihop relaying of data among UTs and BSs or other UTs can reduce high energy consumption needed for direct transmission between distant UT and BS, since communication among relaying nodes can be realised with lower Tx powers. Local content caching provides a way to better exploit the UTs data storage in 5G networks and enables power consumption and backhaul loads reduction through optimal decisions related to what content to cache and at which location (Table 1). Although active user cooperation offers significant advantages in terms of improving SE and EE, further investigations must give an answer on how UTs can be managed to cache, share or relay data for other UTs at the expense of consuming their own energy.

### 3.6. Long-Term Evolution Coexistence with Other Systems in Unlicensed Spectrum

Implementation of LTE-U technology is constantly challenged with the need for simultaneous coexistence of different systems working in unlicensed bands, such as wireless local area network (WLAN) systems and overlay Long-Term Evolution (LTE) systems [12]. Since LTE employs scheduling-based and WLAN contention-based channel access mechanisms, lack of constraints in LTE transmissions may cause permanent interference to WLANs, where the channel is sensed as mostly unavailable [29]. This results in unending backoff times for the WLAN transmitters and poses low EE of the network due to the high energy consumption of the WLAN users lacking the possibility of transmission while waiting on backoff timer expiry. Hence, advanced modifications to resource management become critical for the coexistence of different systems in unlicensed bands, and so far, two methods have been proposed: duty cycling and the listen before talk method. The first one defines periodic turning off and on of the LTE transmitter, without checking the availability of the channel before transmitting, while the second one requires a check of channel occupancy by WLAN systems before the LTE system can start transmission. However, the first method lacks real responsibility of ensuring any transmission time window for WLAN networks since LTE carriers define on-off scheduling, while the second method has degraded performance caused by excessive transmission collisions in case of a huge number of devices contending for the channel (Table 1). Hence, currently there is no broadly accepted protocol that will ensure the harmonious coexistence among systems transmitting in the unlicensed spectrum (LTE-U, WLAN, etc.), and more advanced solutions for alleviating this coexistence issues are jet to be devised.

### 3.7. Energy Harvesting

Wireless power transfer (WPT) known as RF energy harvesting, allows small receivers which are expected to be massively used in 5G use cases like IoT to harvest energy from RF signals which will be received [30,31]. WPT is assumed to be a promising technology for powering a huge number of devices, since harvested energy from RF eliminates the need for powering those devices from an electric gird and also enables battery lifetime extension of mobile, sensor or actuator devices. Although WPT can be fully managed at the receiver side, in the practical implementation of RF energy harvesting, the main challenge is ensuring optimal balance among the harvested energy and the achievable transmission rates. This balance can be realized through the implementation of an approach based on exploiting simultaneous wireless information and power transfer (SWIPT), where the receiver device during reception divides the received signal into two parts, one for energy supply obtained through energy harvesting and the other for information decoding [12]. Another approach known as wireless powered communication network (WPCN) splits information transmission and energy harvesting in time, where wireless devices first harvest energy from received signals and then, by means of harvested energy perform wireless information transmission (WIT). In the case of the first approach, the development of algorithms which will minimize the power losses at the receiver in order to maximise the harvested energy and the achievable throughput must be devised. Regarding the second approach, proliferation of novel solutions which will ensure intelligent selection of WIT and WPT requests for further investigations and improvements (Table 1).

Although RF energy harvesting brings many advantages, the major implementation issue is system performance which is significantly limited by the severe RF signal path loss and consequently low energy conversion efficiency at the position of the energy harvester. One of the possible approaches to system performance improvement is in the implementation of an energy beamforming concept [28]. This concept is based on the transmission of narrow beams through multiple antennas with optimized beamforming vectors, which is fully compatible with M-MIMO and mmWave theologies. Moreover, D2D communications and ultradense networks (UDN) are technologies that contribute to performance improvements of energy harvesting systems. This is because each of these technologies ensures a reduced range among communicating pairs, what reduces the distance for energy transfer and consequently improves WPT efficiency (Table 1). Also, the substation power consumption of electronic circuits during information decoding and channel state information acquisition asks for further attempts in finding new receiver architectures which will consume less power.

Finally, the fact that energy harvesting of co-channel interference can be exploited for ensuring the power supply of receiver devices, gives completely new light on the impact of interference which can become a potential energy source. To exploit interference as an energy source, possible solutions can be based on deliberate artificial interference insertion into communication channels. This approach enables devices to harvest energy in the case of dominant co-channel interference and to decode information in case when this interference diminishes (Table 1). Obviously, more investigations related to such a paradigm shift are needed in future research.

## 4. Research Challenges for Improvements of Data Centres Energy-Efficiency

According to analyses presented in Figure 2b and estimation of data centres (DC) future energy consumption trends presented in Figure 4, increasing trends of DC energy consumption become a major concern. Additionally, DCs continually run at high underutilization due to fragmentation and over-provisioning of resources [32,33], with common utilization levels spanning between 5% and 25% [34,35,36,37]. Besides significant energy waste caused by such low utilisation of DCs which further worsens the energy inefficiency problem, the low DC utilization causes the energy dissipation of other DC ancillary equipment and infrastructure, such as cooling and power supply systems.

**Figure 6:**
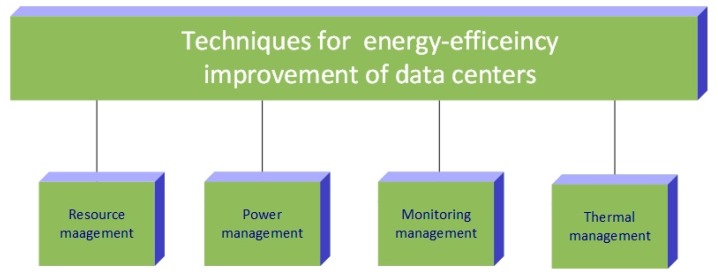
Techniques for energy-efficiency improvement of data centres.

Additionally, authors in [38] analyse green issues related to the processing of the vast volume of information’s characteristic for emerging big data concepts. Analyses address the green challenges related to the three phases of the big data life cycle which are characterized as data generation/acquisition/communications, storage and processing. Also, the study suggests novel green metrics for processing big data in order to accommodate the need for adopting new definitions of green metrics which will correspond to the contemporary big data concept. Although different metrics for expressing DC energy efficiency have been proposed, the widely accepted metric is power usage effectiveness (PUE) defined as [39]:

(2) $$PUE=\frac{P_{TOT}}{P_{IT}},$$

where *P_IT_* is instantaneous power of the IT equipment consumed by the DCs storage, network, servers and monitoring devices (laptops or workstations), and *P_TOT_* is the overall DC instantaneous power consumption which includes the aforementioned *P_IT_* power and instantaneous power consumption of ancillary DC equipment (cooling system, power distribution system, uninterruptable power supply, etc.). In [39], an average value of the present DCs PUE is suggested to be 1.83 and according to the Equation (2), better DC EE means lower PUE and vice versa. Since PUE of present DCs is extremely high, different techniques and approaches for improving EE of DC arise. TheEquation (2) indicates that a better PUE can be accomplished if total DC facility power will be reduced and to this end, research efforts focused on improvement of DC energy-efficiency encompass the following techniques: improvement of DC resource management, increasing DC servers efficiency through power management, developing green DC monitoring and simulations and enhanced thermal management of DC. In Table 2, each of the stated techniques for improvement of DC energy-efficiency with corresponding future research challenges is presented. Also, Figure 6 summarises techniques for energy-efficiency improvement of DCs and an overview of the latest research on green DCs is presented in the next sections.

### 4.1. DC Resource Management

To address resource underutilisation as one of the major DC problems causing an excessive energy consumption, modern servers in DC use the concept of virtualization for presenting the abstraction of many dedicated virtual machines (VMs) or containers executing separate applications (Figure 6) [40]. Hence, optimal migration, allocation and consolidation of DC server resources known as VMs/containers management is an important approach to the improvement of DC resource utilization and energy consumption reduction (Table 2). Generally, VMs/containers management is based on the efficient scheduling of VMs/containers to servers based on satisfying specific performance metrics and resource demands [39]. Although different approaches to VMs/containers management have been proposed [41,42,43,44,45,46,47,48,49,50], the main cause of why the utilization of DC resources still remains ignoble is that DC administrators and owners worry about the potential quality of service (QoS) violations caused by VMs/containers management. Additionally, in multi-tenant DCs, versatile tenants can request different levels of application performance that request heterogeneous resource management algorithms, which further increases its complexity. Hence, algorithms which will optimize DC energy-efficiency through optimal VM/container management and DC right-sizing, while preserving QoS in single and multi-tenant DCs, are at present important research issues. However, improving DC resource utilisation will consequently contribute to the DC energy-efficiency improvements and solutions which will provide efficient resource management policies that require future exploration.

Additionally, traffic engineering is a very efficient concept which enables improvement of DC energy-efficiency (Table 2). It is based on the adaptation of DC traffic paths and network architectures according to DC traffic patterns [51]. To obtain proportionality between DC traffic variations and DC power consumption, different solutions have been proposed based on traffic aggregation and VM/container assignment techniques using virtualization of network functions [52,53,54,55,56,57,58,59,60]. Although network function virtualization promises as an approach in providing EE improvements for deployment and management of the network services, problems such as preserving QoS and lack of accountability for the energy consumption of many implementations such as cloud networking system (for example CloudNaaS) [61] remain unsolved. Hence, finding an appropriate trade-off between network performance and EE is currently a challenging problem that solving requires further research.

Another issue related to DC energy inefficiency is the over-provisioning of DCs power distribution system, which brings high energy costs during idle periods of DCs operation. DC power distribution systems are generally over-provisioned since the deployment of such systems in terms of power capacity is based on satisfying traffic peaks and allowing DC expansions in the future. However, due to the rare occurrence of simultaneous peak power draw across all equipment in DC, power over-subscription is intentionally utilised for enhancing DC power exploitation (Table 2) [34], [61,62,63,64,65,66]. In order to more efficiently utilize the total DC power budget, proposed concepts are based on power capping, power routing and dynamic power shifting among power distribution units (PDUs) and various distributed components. These approaches are performed according to the workload variations and DC power availability. Besides dynamic power shifting, to address the peak power demand issue, a few works have introduced uninterruptible power supplies (UPSs) as an energy consumption saver [67,68,69]. The energy stored in batteries of UPSs is used to provide energy during periods of highest power demand, which results in DC OPEX reductions without performance degradation. Nevertheless, existing works neglect the possibility of inter-DC power scheduling were geographically distributed DCs can also offer opportunities for power distribution (Table 2). Inter-DC power scheduling enables preferment power scheduling to DCs with a larger amount of stored energy by consequence of being allocated closer to the larger sources of energy. Additionally, the low efficiency of UPSs used in DC during low UPS power demand periods, further contributes to the degradation of PUE. Some initial analyses of concept based on the simultaneous UPS and server/VMs consolidation in accordance with the DC workload variations show promising results in terms of improving DC energy-efficiency [70,71]. Still, major challenges related to achieving energy consumption reduction obtained through combining application performance, workload scheduling and power distribution in DC remains. This requests novel and more advanced solutions that can cope with DC power over-provisioning.

The use of renewable energy is another approach to improvement of DC energy efficiency (Table 2). Renewable energy sources such as solar, geothermal or wind energy are investigated for power supply of DC [72]. However, sporadic, unstable and limited nature of renewable energy production significantly determines the use of such green energy for DC power supply. Therefore, the question requesting to be addressed is how to use energy from renewable sources for the power supply of DCs and overcome the associated restrictions. To address intermittent power constraints of renewable energy, most of the previous research activities have been dedicated to the development of solutions in which the DC load had been adapted to follow the variable power supply capacities of renewable energy sources [73,74,75]. However, power supply solutions solely relying on unreliable renewable energy sources can experience unpredictable performance degradation and the most common approach to overcome such challenge is to have a hybrid DC power supply system combining the electrical grid and one or more types of renewable energy for DC power supply [76,77]. Such approaches use weather forecasts and historical data to estimate available renewable energy in the future, with the goal of optimal usage of renewable energy sources. Another challenge in using renewable energy for power supply of DCs is related to attenuation losses caused by the transfer of renewable energy over long distances. To avoid this, several proposed solutions suggest scheduling of server’s workload to multiple DCs located in different geographical locations according to the availability of nearby renewable energy sources [78,79,80,81]. It is shown that such traffic routing based on geographical location can considerably reduce the brown energy consumption, if the energy tariff will be dynamically defined, and the degree of renewable energy usage will depend on the energy pricing model. Hence, future research must offer analyses of beneficial pricing schemes which will encourage DC operators to reduce brown energy consumption.

### 4.2. DC Servers Power Management

#### 4.2.1. Dynamic Frequency and Voltage Scaling

The broadly accepted approach related to the improvements of DC servers power management is based on dynamic frequency and voltage scaling (DFVS) of server components (Table 2). The DFVS as the approach is based on lowering the frequency/voltage of components in order to achieve power savings in periods when the frequency/voltage of server components can be reduced. Due to approximate proportionality between the power consumption and the supply frequency/voltage of different hardware components, the goal is to find an optimal dynamic allocation of frequency/ voltage resources which will minimize the overall power consumption and ensure predefined performance. Vast research results related to the improvement of DC energy efficiency by implementing DFVS management according to individual servers computing and traffic load variations have been presented [82,83,84,85,86,87,88,89,90,91,92,93,94]. Additionally, research efforts on the level of energy-efficiency improvements of the large-scale server′s warehouse through the implementation of DFVS are analysed in [37,95,96]. Since higher frequencies or voltages enable faster execution with the drawback of the increased power consumption, in [95], the optimal power allocation problem related to finding the optimal frequencies/voltages of the server components in a server farm was analysed based on server’s workload. Furthermore, the implementation of an adaptive link rate (ALR) concept on the DCs network level was analysed [89,97,98]. The concept is based on an adaptive selection of speeds of links connecting DC servers in DC communication network. In order to contribute to the DC energy consumption reduction, adaptive adjustment of the Ethernet link data rate according to utilization shows that significant energy savings can be achieved since an Ethernet link can work almost 80% of the time at lower data rates [89]. However, most solutions proposed in the papers related to the DFVS concept, have been focused on power models which are assumed to be ideal. Hence, future research activities should consider models that have more similarities to real systems. More specifically, overhead which is not taken into account is mostly incurred when switching frequency or voltage speeds took place, because the central processing unit (CPU) must stop during these changes. Also, frequent changes in frequency/voltage speed can have a negative effect on CPU lifetime and a challenging issue is how to include these facts in performance analyses of practically implemented systems.

#### 4.2.2. On/Off Server and Component Switching

Another approach related to the DC server′s power management is based on the server components activity scaling, which envisions the transition of server components (such as server CPUs, memory, etc.) during idle traffic and computing periods into sleep or low-power standby mode (Table 2). The challenge is to decide when sufficiently long idle periods exist that enable component (CPU) activity state switching, while the cost for transitioning from or into the low power consumption state will not outweigh the costs incurred by this transition and will satisfy the workload demand. Analyses of the challenge of scheduling the power consumption in a two (sleep and active) states are presented in [99], and different studies extend analyses with multiple stets in [100,101]. Generally, components (such as CPU) state transition energies were assumed to be additive [100,102]. By taking into account different assumptions related to CPU state transition energy, different CPU scheduling algorithms in the case of single and multi-processor environments were proposed [104,105].

Additionally, a number of different studies proposed energy proportional computing for large hosting DCs [105,106,107,108,109,110], which are based on the concept of dynamic activation and deactivation of DC servers proportional to the DC workload demand (Table 2). Through such energy-aware provisioning, the server load is directed to the minimal active set of servers in DC and reduction of the server power consumption by 29% for characteristic web-based load is reported in [111]. Nevertheless, the novel approaches to further optimize DC energy consumption need to be devised. More specifically, in widely accepted parallel scheduling of jobs to different CPUs of servers which number is fixed, the scheduler decides about jobs that will be processed on CPUs and make a decision at any given time about the speed of each CPU. But, DCs operate on a different concept in which on-demand activation or deactivation of servers must be achieved. Hence, such DCs properties impose the development of new algorithms related to improving PUE while satisfying DC scalability and efficiency. Also, the power consumed, and latency generated during the rebooting of servers means that the effects of on/off server switching or DC networking device switching must be taken into account.

Furthermore, power-down mechanisms based on the concept of aggregating and redirecting network traffic on a few network devices which remain active are proposed in [106,107,108,109]. However, DC network architectures often ensure many communication paths between servers. This imposes the challenge of how to effectively control power consumption in DC networks and requests deeper investigation which will offer novel topologies and designs of DC networks, while satisfying demands for the network delay, congestion, loss of packets and throughput in those networks.

#### 4.2.3. Hybrid DFVS and On/Off Server Switching

Another approach for improvement of DC server power management is based on hybrid concepts that exploit both DFVS scaling and servers or server components switching models. This hybrid approach is seen as a promising approach that can bring further improvements in DC energy-efficiency. This approach considers accelerating the processing tasks of server or server component activity, which results in longer idle periods, during which devices can be in the sleep or shut-down mode. Longer idle periods then give a higher contribution to the energy savings. The first theoretical analysis with an algorithm enabling combining system sleep mode for idle workload periods and DFVS during task processing periods are presented in [110]. In subsequent studies [111,112,113,114], improved algorithms were presented, some of which enable on-line scheduling and have low complexity orders. To enhance energy savings at the level of complete hosting DCs, in [116] a framework allowing the implementation of both approaches is introduced, while in [116], the authors considered the power consumption reduction in geographically distributed DCs. Hybrid techniques for improving the energy efficiency of network elements in DCs are used in studies [117,118]. In [117], to reduce network energy consumption, hybrid technology is implemented based on adjusting the rate of network operators to the real workload. Furthermore, in [118], the authors formulated an approach for online traffic management which reallocates the computing demands among a multitude of paths while optimizing energy consumption. Although both approaches report some energy savings, further investigations related to the implementation of the hybrid approach must take place.

Additionally, current results mostly focus on DC environments having servers with multiple homogeneous processors. Nevertheless, it is also important to take into account the DC server′s heterogeneity, since the servers in DC mostly differ among themselves in terms of computing and hardware performance. Hence, results for heterogeneous environments in terms of the design of energy-efficient algorithms that can combine different DC power management methods are currently missing.

### 4.3. DC Simulation and Monitoring Management

Effective monitoring of DCs enable detecting the traces and tracks of thermal emission, power distribution and energy consumption for individual DCs equipment. Collected data can be used for the implementation of intelligent mechanisms based on which, the DC energy efficiency can be increased. Different DC monitoring services have been presented in [119,120]. Based on the collected data related to the VM application workloads, the resource utilization and power usage, DC online monitoring service presented in [119] enable a better understanding of the DC temperature behaviour and energy consumption. Developed monitoring solution also helps in consolidating the VM workload, which contributes to the significant energy savings. In [120], a monitoring solution based on request-tracing concept was implemented for determining energy inefficiencies in multi-tier DCs. The solution is based on collecting the resource consumption of respective requests and analyses of the characteristics of every DC tier. This further enables insights into the main causes of energy inefficiency of DCs and gives an opportunity to devise efficient power-saving methods for multitier applications.

As an example, green storage initiative (GSI) of the Storage Networking Industry Association (SNIA) works on forming a global standard for defining energy efficiency metrics of storage products working in the DC environment [121]. The proposed methodology enables the standardized and uniform method to grade the power efficiency of commercial (file, block, converged, object, etc.) storage in idle and active working states. This enables selecting the type of storage which best suits DC owner goals with the lowest power consumption contribution. This also motivates manufacturers to develop more energy-efficient storage devices since its energy efficiency can be compared among vendors.

Although monitoring of green metrics offers diagnosing of DCs energy inefficiencies, the development of monitoring tools has not been in the main research focus so far. The main obstacles in the realization of efficient DC green metrics monitoring are the availability of communication resources in DCs and a huge number of VMs and containers hosted on a large number of servers. Thus, future research activities need to be focused on solving the key research question related to the minimization of the costs incurred during collecting green DC metrics in a centralised or distributed manner, while guaranteeing monitoring accuracy.

Another approach to improvement of DC energy efficiency is based on simulation of DCs activity by means of developed simulation tools that enable understanding and identification of the design challenges that are crucial to DC energy efficiency. In this regard, different simulation platforms such as SimWare [121], GDCSim [122], GreenCloud [123] and EEFSim [124] are proposed. SimWare simulator [121] enables evaluation of the DC energy-saving policies and examination of mechanical functionalities such as management of airflow, cooling strategies and server placement. GDCSim proposed in [122] enables the iterative design of green DCs configurations for specific purposes, such as DFVS for power management, CPU sleep-state transitions and characterisation of thermal behaviour. GreenCloud [123] is a packet exchange simulator for energy-aware analyses of cloud DCs, which can be used for capturing the energy consumption of versatile DC elements such as switches, links, servers, as well as packet-based communication patterns. EEFSim [124] reproduce the behaviour of a real cloud DC and enables the possibility to examine the power consumption of different migration and scheduling policies with VMs. Nevertheless, the main drawbacks of these simulators are that each of them is very specific and dedicated only to certain functions or components of DC equipment such as CPU, VM, cooling, etc. Therefore, the task of designing a comprehensive system-oriented DC simulator that will integrate all DC components, such as the memory, CPU, cache, disks, input/output components and communication network is still an open task which requests addressing.

### 4.4. DC Thermal Management

Since typical DC hosts thousands of servers and communication network devices, it is reported in [125,126] that up to one-half of the total DC costs are spent on the cooling. These trends will be further contributed with previously presented servers′ virtualization and consolidation techniques. These techniques increase processor utilization rates, which consequently contribute to the increase of thermal dissipation. Additionally, a combination of the abovementioned issues with trends characterised with server’s concentration and high-density computing (realised through usage of many multi-core processors in single chasses), will raise the problem of thermal control as one of the most critical issues in deploying green DCs.

To cope with such a problem, in [127] the optimization of the DC cooling delivery is based on full control of the DC environment through collecting different DC attributes such as data aggregation, variable air conditioning and distributed sensing. Although this concept reports energy savings of up to 50%, another approach based on exploiting thermal energy storage (TES) tanks for the reduction of DC power is presented in [128]. In this concept, up to 28% in OPEX reduction is reported since this approach is based on TES storage of cold water or ice which are exploited as a supplement to the chillers used for cooling DCs and for heat exchange during peak power periods. Additionally, a very promising research field which can improve DC thermal management is based on DC workload migration and assignment among servers, in order to achieve thermal balance [129,130,131]. The general idea is dedicated to the development of algorithms for load scheduling based on temperature variations, which can reduce the energy consumption of the infrastructure dedicated to DC cooling [129]. The approach proposed is based on the dynamic transfer of server’s workload from ″warmed″ servers and increasing the workload on remaining ″colder″ servers. The approach in [130] uses periodic temperature monitoring and server utilization for scheduling requests according to the DC workload weights. Also, in [131], a data-centric model dedicated to minimization of energy costs for DC cooling is developed based on dynamic file allocation in an energy and thermal-aware manner. The proposed model is developed by means of known data-semantics, cluster information and server-profile. Proposed approaches show possible energy savings between 20% and 42%. Additionally, in [132], the challenge of temperature-aware workload distribution in geo-distributed DCs is shown.

A completely different approach is based on efforts related to the reduction of the costs imposed by cooling, if the higher temperature can be sustained in DC. Basically, the concept is based on increasing temperature setting by only a few degrees, which results in an energy consumption reduction of 2%–5% [133]. However, the temperature increase of the servers and other equipment in DCs can contribute to a shortening of the DC equipment lifetime, which further contributes to the increase of capital expenditures (CAPEX) costs. Some initial studies related to the analyses of hardware (storage/memory/server) reliability and server performance presented in [134] show that in order to save energy, the DC could work at hotter temperatures than current ones, while negative effects on system reliability and performance can be partially limited. Still, better analyses of how temperature raise in DC can affect DC systems are needed and this field remains an open research topic.

Some other approaches which can offer the possibility of DCs to operate at higher temperatures are related to the development of new temperature-resistant hardware components. However, such components are still in their infancy and novel temperature-resistant chip and hardware solutions should be developed.

## 5. A Review of Articles for Special Issue on Green, Energy-Efficient and Sustainable Networks

The paper [135] analyses the influence of the node speed on the throughput and energy provision in an IoT network, where wireless charging stations (WCSs) are deployed to recharge IoT nodes while data transfer among nodes is limited by their abrupt links as well as the amounts of residual energy. To optimize node throughput and energy depletion of IoT nodes in such network based on wireless power transfer (WPT), authors propose a two-dimensional model based on Markov chains where the first state dimension represents the span to the closest WCS normalized with speed of nodes, while the second one represents residual energy of the node. Obtained results show that to enhance wireless charging efficiency, charging opportunity must be prioritized by WCSs based on a speed of IoT nodes, for which battery capacity can be minimized if the speed of nodes can be predicted. Also, if the same throughput must be ensured, it is shown that a lower number of WCSs per node can gain appropriate WPT to all nodes in the area of high mobility, while a larger number of WCSs per IoT node are needed in areas of low mobility.

The next paper [136] tackles the problem of improving the energy-efficiency of software-defined networking (SDN) equipment based on the concept of traffic aggregation on links between two switches. In the paper, authors present different traffic allocation algorithms for SDN applications, which enable aggregation of the traffic flows to a few ports of the Ethernet links bundle in accordance with the traffic variations. Proposed allocation algorithms are validated in terms of packet losses, energy-efficiency and delay of packs. Obtained results show that the implementation of equipment with SDN capabilities can reduce energy consumption when Ethernet link bundles are used for up to 50%, without the necessity of changing devices firmware. Also, improvements of the two previous algorithms dedicated to offering a low-latency service for data traffic with strict requirements in terms of QoS and sustained energy consumption are proposed. According to the shown results, the algorithms can ensure the service which requests a low-delay of some orders of magnitude to time-sensitive traffic.

In [137], image compressive sensing is analysed as a potential image sensing approach that can satisfy green IoT demands in terms of finding optimal storage and data organization format suitable for sensors with limited power and bandwidth availability. The layer, patch and raster structure are proposed as three promising measurement schemes that differ in approaches related to storing and packaging of sensing measurements within an image. It is shown that each of the three proposed measurement structures restrains the image blocking artefacts and eliminate high memory requirements and huge computation complexity during image sensing and recovery. However, the layer structure shows the best results in terms of possible green IoT implementation since it has good rate and time-distortion performances and offers better visual quality than other structures.

Work [138] addresses a lack of models for energy-efficient malware detection based on gaining knowledge about devices in an IoT environment with the Android operating system (OS). In the paper, adversarial samples vulnerability of learning-based malware detection models is tackled through the development of an automated testing framework that performs security analyses for IoT devices. In order to find an appropriate fitness function that can produce the corresponding sample without impacting the characteristics of the application, authors introduce generic algorithms and specific technical enhancements built-in proposed testing framework. Obtained results show that black-box testing of the system can be done by the proposed test framework, which can create effective samples with a rate of success equal to almost 100% for the application on IoT devices with Android OS.

To eliminate drawbacks of authentication based on cipher approaches that are impacted by the large expenditures and energy constraints of smart devices, authors in [139] proposed a clustering-based physical-layer authentication scheme (CPAS) for systems with asymmetric resources in the mobile edge computing (MEC) environment. To ensure two-way authentication among edge devices and terminals, CPAS as cross-layer secure authentication merge symmetric cipher and clustering with information related to the wireless channel. Theoretical analysis of developed CPAS approach shows that CPAS can be robust to replay, spoofing and integer attacks, while experimental results show that CPAS decreases the data frame loss rate and increase the overall success rate of access authentication, without enlarging authentication latencies. Therefore, the proposed scheme reduces the complexity of resource asymmetric authentication scenarios for the edge computing systems, which contributes to the reduction of power consumption during the authentication phase.

In [140], the problem of the inter-tier interference mitigation in two-tier HetNets composed of pico-cells and underlying macro-cells has been considered. First, the near-optimal values of almost blank subframes (ABS) power reduction factor and pico-cell range expansion (CRE) bias are gained by an algorithm which uses equivalence relation between ABS and CRE for a given pico-cell base station (PBS) density. Also, by means of a linear search method, PBS density is optimized with the known factor of power reduction and constant pico-CRE bias. Lastly, to maximize network energy-efficiency of two-tier HetNets impacted with further-enhanced inter-cell interference, authors propose a heuristic algorithm for joint optimization of ABS power reduction factor, PBS density and pico-CRE bias. Results obtained through numerical simulations show that the proposed heuristic algorithm with a low complexity of computation can update the HetNets energy efficiency.

The study presented in [141] extends preceding works that use the social behaviour of the mobile users to adapt the transmission speeds of messages used for the peer discovery in D2D networks under the user equipment′s (UEs) power consumption constraint. The authors introduce a three-phase energy-ratio rate decision (ERRD) algorithm, which in the first phase schedules the power budget of the network among the UEs based on their social ratios and in the second phase, based on harvested energy, allocates power quantum of each UE. Finally, in the third phase of the ERRD algorithm, the UEs beacon transmission intensities are adjusted according to their designated quantum of power. Adjusting is performed under the limitation that the overall power scheduled to the UEs cannot be above the power quanta of the network budget. Results obtained through simulations of ERRD performance show that the proposed algorithm outperforms the previously-reported algorithm by 8% and 190% on the peer discovery ratio, for a budget having the power of 20 and 1 W, respectively.

In paper [142], in order to improve the secure operation of industrial wireless sensor networks (IWSNs), a physical layer authentication based on deep learning is presented. Three different authentication methods for sensor nodes, more specifically the deep neural network (DNN), the convolutional neural network (CNN) and convolution pre-processing neural network (CPNN) have been used to deploy the PHY-layer authentication in IWSNs. According to simulation results obtained during the evaluation of algorithms performance, each algorithm can authenticate multiple nodes simultaneously trough lightweight authentication. However, the CPNN-based sensor nodes’ authentication method has the best trade-off between the shortening of algorithm authentication performance and the minimal training time of the algorithm.

Paper [143] investigates the mobile directional charging vehicle (DCV) efficiency optimization in rechargeable wireless sensor networks (RWSN), through the implementation of wireless power transfer (WPT) on continuously working sensors. Authors initially design an approximation algorithm to define positions and charging orientations of the docking spots, with the constraints of maximizing the charging coverage utility and minimizing the total number of the DCVs docking spots. Then, an optimization of the DCVs energy charging is performed based on the developed moving path planning algorithm for the DCVs. Based on theoretical analyses and comprehensive simulation experiments, for the case of sparse networks, authors present that the efficiency of energy charging of the proposed DCV concept is better than those based on a model using the omnidirectional energy charging.

Authors in the next accepted work [144] have analysed the problem of green networking from the sustainability point of view. Besides energy-aware routing, authors propose pollution-aware routing with new metrics like the percentage of non-renewable energy usage and CO_2_ emission factor. The proposed algorithm provides optimal control and data planes for these metric types and enables different routers scheduling and link bandwidth adaptations, while ensuring scheduling and adoption priority according to traffic demand requirements. The impact of the proposed algorithm enabling green routing was assessed for three different metrics. Obtained results show that the proposed pollution-aware routing algorithm can reduce CO_2_ emissions for 20% and 36%, if compared with energy-based and shortest path routing, respectively.

A relaying system based on non-orthogonal multiple access (NOMA) in downlink transmission with the best amplify-and-forward radio-frequency energy harvesting relay was analysed in work [145]. Analyses are performed for a source node that exchanges information in parallel with multiple users and for the Rayleigh fading conditions lacking perfect RF channel state information. For such a system, authors develop expressions for the outage probability (OP), the optimal duration of energy harvesting which minimizes the OP and the ergodic capacities of each user. Based on numerical results obtained for the equal setting of parameters, the ergodic capacity of the whole system and overall performance of the proposed NOMA relaying system outperforms an equal system with the orthogonal-multiple-access (OMA) relaying.

Authors in [146] analyse the influence of using single and multiple relays on energy-efficiency and throughput of Long-Term Evolution-Advanced (LTE-A) networks, for different resource block (RB) allocation schemes. Energy-efficiency analyses for a single relay scenario is performed for the maximum throughput (MT) bisection-based power allocation (BOPA) algorithm, an alternating MT with proportional fairness (abbreviated SAMM) BOPA algorithm and SAMM equal power RB algorithm. Simulation results show that the SAMM BOPA algorithm ensures the best energy-efficiency, while SAMM equal power algorithm provides the best fairness. For a multiple relay scenario, a two-step neural network (NN) algorithm (SAMM NN) is introduced. Algorithm exploits BOPA supervised learning for power scheduling and SAMM unsupervised learning for scheduling of RBs. Results obtained for multiple relays scenario shows that SAMM NN algorithm achieves better energy-efficiency in comparison with SAMM equal power and SAMM BOPA algorithms.

Article [147] analyses the radio frequency fingerprinting identification (RFFID) approach dedicated to ensuring authentications for a high number of energy-limited user terminals working in the MEC environment. The proposed scheme combines a two-layer model with the use of non-encryption RFFID for IoT terminals. In the first layer, the MEC devices perform access authentication after signal detection and RF fingerprint features extraction with database storage of collected features. In the second layer, implementation of machine learning algorithms through collected learning features and generated decision models is done in the distant cloud, which improves the speed of authentication. Through extensive simulations performed for scenario based on IoT implementation, the gained results indicate that the approach proposed in [147] can achieve lower device energy depletion and better recognition rate than the traditional RFFID method based on wavelet features.

Paper [148] tackles the problem of scheduling consumer′s requirements and the achievable electricity provision from renewable sources through the demand-response (DR) model. The proposed DR model is centralised via the data collector called the ″aggregator″ which schedules consumer’s requirements for instantaneous power supply and supplied electricity from renewable energy sources in a home environment monitored through the implementation of IoT applications. Results of the proposed algorithm evaluation confirmed algorithm feasible costs of computation in different scenarios of consumer′s behaviour and versatile communities and households. Also, it is shown that the energy reallocation costs are mostly impacted by a consumer′s demand timeframe flexibility and a number of appliances.

In [149], the challenge of optimizing the power consumption of network devices in DC by means of energy-aware traffic engineering was addressed. The authors propose an optimization approach based on a mixed-integer programming algorithm, which minimizes network devices′ energy consumption according to traffic load variations. The proposed approach was verified through simulations of versatile DC network topologies and obtained results demonstrate clear benefits in terms of DC power consumption reduction for different traffic volumes and DC network sizes. Furthermore, the proposed approach can be deployed as an implementation in the SDN paradigm, and therefore, it can be used in real practical implementations.

In the review paper [150], the energy efficiency of the radio access and core parts of the 5G networks are surveyed, and open issues and challenges related to the achievement of green cellular access networks are discussed. An overview of techniques for energy-efficiency improvement at the BS level encompasses next techniques for 5G networks: dynamic on/off cell switching, interference-aware energy efficiency control in UDNs, energy efficiency enhancement of BSs with radio resource control, connection management for 5G new radio, and energy-efficient cache-enabled BSs. Further analyses have been dedicated to the review of techniques for energy-efficiency enhancement at the 5G network level which includes energy-efficient: resource sharing, resource allocation in NOMA, outdoor–indoor communications and virtualization techniques. Additionally, authors perform a survey of SDN technology for improving energy-efficiency which considers energy monitoring and management in 5G with included backhaul and fronthaul and energy savings approach based on the utility of sleep mode. Finally, the authors give an overview of techniques based on machine learning for energy-efficiency improvement of 5G networks.

## 6. Conclusions

Just a decade ago, the energy consumption of ICT devices and systems have been postponed in network and device design. However, a point where energy consumption optimisation of ICT devices and systems have become the new frontier for competitive differentiation and innovation is reached. Having energy-efficient ICT systems and devices is no longer a nice-to-have feature, but the mandatory requirement for the networks of the upcoming digital age. This is confirmed in papers accepted for publication in the Special Issue on the green, energy-efficient and sustainable networks, which overview in terms of addressed topics and obtained outcomes are presented in this paper. Additionally, estimations and analyses of energy costs and CO_2_ emissions for different ICT systems in the period ranging up to the year 2030 are surveyed. Presented analyses confirm that ICT systems are at a critical point regarding current and future energy consumption of telecommunication networks, DCs and user-related devices. According to the presented estimations, current technology improvements of different ICT systems are not sufficient to keep up with the increasing energy costs and CO_2_ emissions. This is elaborated in this paper for the case of wireless networks and DCs, which energy consumption and CO_2_ emissions have the highest increase and contribution to the overall ICT energy consumption. This motivates the deeper investigation of technologies and concepts which can contribute to the improvement of energy-efficiency of these ICT sectors. As presented in this work, for the wireless networks, possible technologies that are analysed in this context include millimetre-wave communications, long term evolution in unlicensed spectrum, ultra-dense heterogeneous networks, device-to-device communications and massive multiple-input multiple-output communication. Additionally, DC resource management, DCs power management, green DC monitoring and simulation and thermal management in DCs are discussed as possible options for improvement of DCs power usage efficiency. Although each of analysed techniques and concepts can bring some energy-efficiency improvements in the corresponding area of implementation, comprehensive analyses presented in this paper shows that there is no single technology or concept which will bring energy-efficient improvements to the whole ICT sector. Hence, to achieve more power-efficient and greener ICT systems in the future, the combination of different technologies and concepts in wired, wireless and DC part of communication networks with novel solutions for energy-efficiency improvements of user-related and sensor devices must be devised. Only such an approach can result in a synergetic effect which will keep energy consumption and CO_2_ emissions of ICT systems at the lowest possible levels.

## Figures and Tables

**Table 1:** Technologies for energy efficiency improvements of wireless networks and future research challenges.

Technology	Energy-Efficiency Improvement Area	Future Research Challenges for EE Improvements
Ultra-dense HetNets [13,14,15,16,17,18,19,20]	Network design with decupled data and signalling	Development of effective algorithms for the management of signalling and data decupling
	Network design with BS on/off switching	Development of effective radio resource management algorithms for efficient BS activations and deactivations
	Network design with inter-cell interference mitigation	Development of efficient inter-cell interference management schemes
M-MIMO [12,14,21]	Design of energy-efficient antenna selection	Finding algorithms for the selection of an optimal number of antennas in M-MIMO systems
	Energy-efficient hardware design	Finding novel hardware designs for multi-antenna placement in UTs
	Energy-efficient design of pilot tones	Finding algorithms for reducing the energy consumption of pilot tome transmission
mmWave communications [12,22,23,24]	Energy-aware transceiver designs	Finding optimal hybrid control of RF transceiver architectures and antenna designs
	Energy-efficient analogue-to-digital converters design	Finding optimal analogy-to-digital converters in terms of sampling rate resolution
Renewable energy sources [25,26,27]	System design which exploits renewable energy and energy cooperation	Solutions for estimation of optimal renewable energy sources for BS sites
	System design which exploits energy cooperation	Development of systems enabling surplus power transfer among BS sites
	Design of BS site with efficient energy flows management	Development of an optimal algorithm for energy flow management on sites with renewable energy sources
D2D communications [12,28]	Network design based on the hybrid overlay and underlay communication	Development of algorithms for switching among underlay (assigned spectrum portion) and overlay (unassigned spectrum portion) communication designs
	System design which enables active users’ cooperation	Development of algorithms for caching, sharing or relaying data with minimal UTs energy consumption
LTE-U coexistence with other systems [12,29]	Design of channel allocation protocols	Finding optimal protocol for RF channel scheduling among different systems in an unlicensed band
Energy harvesting [30,31]	Design of highly efficient energy harvesting systems	Development of algorithms for optimally balance between energy harvesting and data transmission
	Design of system which reduces energy conversion inefficiency	Development of systems based on energy beamforming, D2D and HetNets communications with more energy-efficient receivers
	Development of systems which exploit interference in wireless networks	Development of systems which optimally exploits interference signals for energy harvesting

**Table 2:** Technologies for EE improvements in data centres and future research challenges.

Technology	Energy-Efficiency Improvement Area	Future Research Challenges for EE Improvements
DC resource management [36,37,38,39,40,41,42,43,44,45,46,47,48,49,50,51,52,53,54,55,56,57,58,59,60,61,62,63,64,65,66,67,68,69,70,71,72,73,74,75,76,77,78,79,80,81]	Energy-aware VM/containers assignment in DCs	Finding an optimal algorithm for the implementation of energy-efficient VM/containers management
	Energy-aware DCs network traffic engineering	Development of algorithms for energy-efficient adaptation of DC traffic paths and network architectures
	Energy-efficient power distribution in DCs	Design of energy-aware solutions for intra and inter DC workload scheduling and power distribution
	Usage of renewable energy for DC power supply	Finding solutions for optimal control of DC power supply form renewable energy and implementation of stimulating energy pricing models
DC servers power management [82,83,84,85,86,87,88,89,90,91,92,93,94,95,96,97,98]	Energy-aware DFVS scaling of server components	Finding optimal frequency/voltage and link speed scaling solutions for minimization of the DC power consumption
	Energy-aware server/server component activity scheduling	Development of novel energy-efficient algorithms for on/off server or server components switching
	Energy-efficient hybrid (DFVS and component activity switching) solutions	Development of algorithms which combine DVFS and on/off server or server components switching
DC monitoring and simulation management [99,100,101,102,103,104,105,106,107,108,109,110,111,112,113,114,115,116,117,118,119,120,121,122,123,124,125,126]	Green DC monitoring	Development of novel DC monitoring tools which will enable analyses of green metrics
	Green DC simulators	Design of a system-oriented DC simulator for concurrent performance simulation of different DC elements
DC thermal management [127,128,129,130,131,132,133,134]	Energy-efficient cooling and workload distribution	Development of temperature-aware DC workload assignment algorithms
	DC management system which improves temperature to reliability trade-off	Design of novel temperature-resistant components for DCs with an increased average temperature
